# Could −79 °C Spray-Type Cryotherapy Be an Effective Monotherapy for the Treatment of Keloid?

**DOI:** 10.3390/ijms18122536

**Published:** 2017-11-26

**Authors:** Tae Hwan Park, Hyeon-Ju Cho, Jang Won Lee, Chan Woo Kim, Yosep Chong, Choong Hyun Chang, Kyung-Soon Park

**Affiliations:** 1 Department of Plastic and Reconstructive Surgery, CHA Bundang Medical Center, CHA University, Seongnam 13496, Korea; RA22211@chamc.co.kr (J.W.L.); A176004@chamc.co.kr (C.W.K.); 2 Department of Biomedical Science, College of Life Science, CHA University, Seongnam 13488, Korea; whguswn94@chauniv.ac.kr; 3 Department of Hospital Pathology, College of Medicine, The Catholic University of Korea, Seoul 06591, Korea; roja30@hanmail.net; 4 Department of Plastic and Reconstructive Surgery, Kangbuk Samsung Hospital, Sungkyunkwan University School of Medicine, Seoul 03181, Korea; eppeene@hanmail.net

**Keywords:** keloid, cryotherapy, fibrosis, matrix metallopeptidase 9, transforming growth factor β 1

## Abstract

Cryotherapy has been regarded as an effective modality for the treatment of keloids, and the spray-type device is one of the novel cryotherapeutic units. However, the biological mechanisms and therapeutic effects of this technique are incompletely studied. We evaluated the clinical efficacy of our cryotherapy protocol with molecular and pathologic evidence for the treatment of keloids. We evenly split each of ten keloid lesions into a non-treated (C−) and treated (C+) area; the C+ area was subjected to two freeze-thaw cycles of spray-type cryotherapy using −79 °C spray-type CryoPen™. This treatment was repeated after an interval of two weeks. The proliferation and migration abilities of the fibroblasts isolated from the dermis under the cryotherapy-treated or untreated keloid tissues (at least 5 mm deep) were compared and pathologic findings of the full layer were evaluated. Molecular analysis revealed that the number of dermal fibroblasts was significantly higher in C+ group as compared with C− group. The dermal fibroblasts from C+ group showed more than two-fold increase in the migration ability as compared with the fibroblasts from C− group. The expression of matrix metallopeptidase 9 was increased by more than two-fold and a significant increase in transforming growth factor beta 1 expression and Smad2/3 phosphorylation level was observed in C+ group. C+ group showed more extensive lymphoplasmacytic infiltration with thicker fibrosis and occasional “proliferating core collagen” as compared with C− group. Thus, −79 °C spray-type cryotherapy is ineffective as a monotherapy and should be used in combination with intralesional corticosteroids or botulinum toxin A for favourable outcomes in the treatment of thick keloids.

## 1. Introduction

Patients with keloids typically present with pruritus, pain, ulceration, secondary infection, restricted motion, and psychological symptoms due to cosmetic disfigurement [1]. Given their intractable nature and high recurrence rate, keloids are a great burden to the patients, physicians, scientists, and society and are associated with physical, aesthetic, and social complaints [2,3]. Numerous treatment methods, including cryotherapy, intralesional injection, laser treatment, pressure therapy, radiation, and topical treatment have been proposed for keloids [4]. Cryotherapy has been widely used for years and is regarded as an effective modality for the treatment of keloids; several techniques and devices related to cryotherapy have been developed in the clinical practice [5,6].

In 1982, Shepherd and Dawber first described cryotherapy as an optimal treatment options for keloid scars [7]. External cryotherapy, contact cryotherapy with spray technique, is an efficient and effective method for clinical use and has been performed over the past few years; however, numerous side effects such as edema, swelling, blister formation, or oozing are inevitably encountered during the wound-healing process [8].

Intralesional cryotherapy was designed in 1993 by Weshahy [9] and involves intralesional application of liquid nitrogen with a needle to destroy the core of the keloid scars. It is an effective method, wherein the scar tissue is frozen from the inside and responds well to a single use. The recurrence rate is reduced with intralesional cryotherapy and a favourable reduction in the scar volume was observed as opposed to the external cryotherapy. However, it usually requires local anaesthesia and patients often complain about severe pain and bleeding, due to its relatively invasive nature. In addition, it requires general anaesthesia in cases of large keloids, leading to increased morbidity, hospital visits, and medical cost. Furthermore, the application of intralesional cryotherapy is difficult for wide superficial keloids. Considering the recent trends of minimally invasive techniques, many physicians use technologically advanced spray-type cryotherapy such as −79 °C hand-held spray to treat warts and other skin lesions. In recent years, this spray has been used for keloid treatment, as it eliminates the need for local anaesthesia. As a consequence, it offers less pain and discomfort and reduced psychological burden as compared with the conventional cryotherapy device using −196 °C or intralesional spray. However, the underlying mechanisms and therapeutic effects of this technique are questionable. Therefore, we employed −79 °C spray-type CryoPen™ in this study.

Matrix metallopeptidase 9 (MMP9) has been implicated in the keloid pathophysiology, due to its gelatinase activity. The enzyme activity of MMP9 induces extracellular remodelling through the degradation of gelatins (collagens) and matrix-associated substrates such as aggrecan and elastin [10,11]. In addition, MMP9 converts various cytokines and chemokines into their more active forms [12,13,14]. Though the role of MMP9 in keloid development is incompletely studied, several reports reveal its active role in the development of keloid lesions. MMP9 is significantly overexpressed in keloid-derived fibroblasts, especially at the margins of the keloid wound [15]. Transforming growth factor beta 1 (TGF-β1)-mediated up-regulation of microRNA-21 increases the expression of MMP9 in keloid fibroblasts, wherein it promotes fibroblast proliferation and transdifferentiation [16].

In this study, we evaluated the clinical efficacy of −79 °C spray-type CryoPen™ with molecular and pathologic evidence for the treatment of keloids.

## 2. Results

### 2.1. The Schematic Illustration of Tissue Sampling for Pathologic and Molecular Study

**Figure 1:**
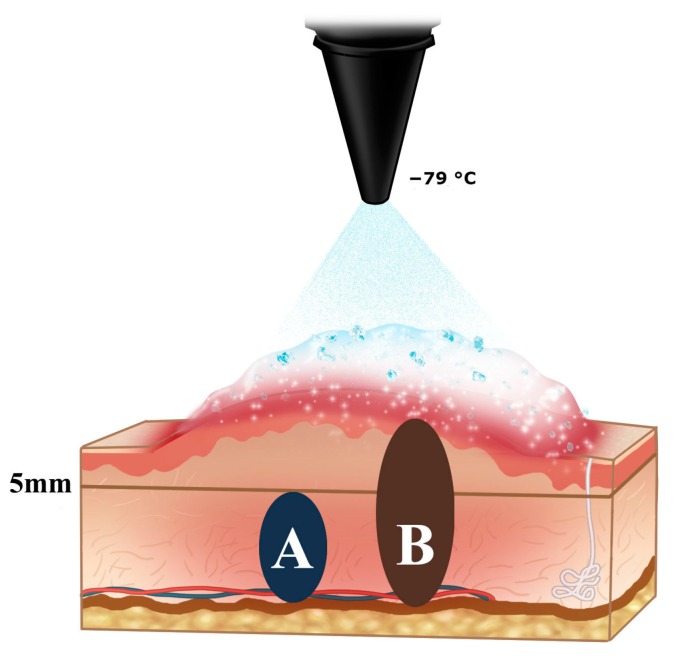
Schematic illustration of our tissue sampling for pathologic and molecular study. (A) The dermal keloid (3 mm thick and at least 5 mm deep) from the epidermis was used for primary cell culture, (B) while the adjacent full layer from the epidermis to reticular dermis was used for pathologic examination.

A total of six patients with ten keloids were treated with −79 °C spray-type CryoPen™. Of these, two patients displayed multiple lesions (four and two), which were treated with cryotherapy. The mean age of the patients was 29 years (range: 17–56) and the duration of scar ranged from 0.5 to 30 years. Four keloids were of Fitzpatrick skin type III–IV, while six keloids were of skin type I–II. The locations of scars were chest wall, upper arm, pubis, axilla, shoulder, trochanter, ear lobule, and helix (Table 1). We selected two cases for the molecular and pathologic study (Case #1 and #5). The dermal keloid (3 mm thick and at least 5 mm deep) from the epidermis was used for primary cell culture and the adjacent full layer from the epidermis to reticular dermis was used for pathologic examination (Figure 1).

### 2.2. Keloids Treated with −79 °C Spray-Type CryoPen™ Rapidly Reoccurred in Clinical Trials

**Figure 2:**
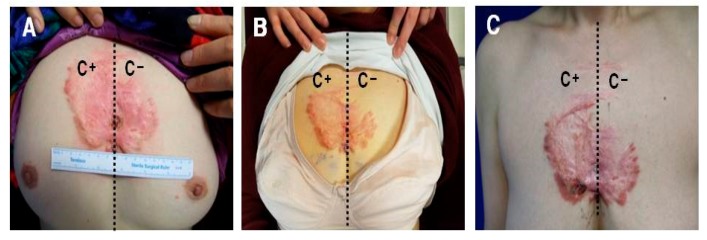

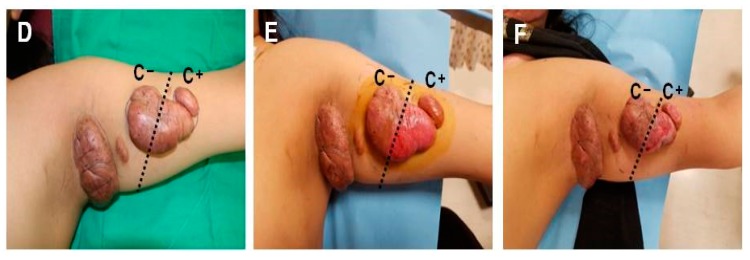
Clinical cases of keloids treated with −79 °C spray type CryoPen™. (**A**) A 56-year-old female patient with anterior chest keloid was presented with recurrent central ulceration, pruritus, and intermittent pain; (**B**) Split therapy was performed such that the right half was treated with cryotherapy, while the remaining left half was left untreated. The therapeutic effect was noticed 1 week following four sessions of cryotherapy in terms of improvement in the texture, softness, and redness accompanied with a slight shrinkage of the right treated half; (**C**) Three weeks after the four sessions of cryotherapy, the lesion reverted to its original status unless it was subjected to repeated cryotherapy; some skin erosion was observed in the treated half (small white arrow). Keloid excision and full thickness skin grafting from inguinal area were planned; (**D**) A 21-year-old female patient was presented with spontaneous keloids of the upper arm and axilla; (**E**) Split therapy was performed such that the lower half was treated with cryotherapy, while the upper half was left untreated. The therapeutic effect was evaluated after four sessions of cryotherapy in terms of reduction in the volume or shrinkage of keloid. Cryotherapy was ineffective and caused skin erosion at the site of cryotherapy; no volume reduction or shrinkage (small white arrow) was observed; (**F**) Post-treatment appearance of keloid at 3 weeks after final cryotherapy. The erosion was improved with conservative wound management. Keloid excision and primary closure were planned.

As shown in Figure 2A,B, the keloid lesion located at the chest wall responded well to the protocol in terms of redness and hypertrophy and the symptom improved for 2 weeks after cryotherapy. However, the lesion reverted to its original status after 2 weeks unless it was treated with cryotherapy (Figure 2C). However, the cryotherapy was ineffective for severe cases of keloid located at the upper arm and caused skin erosion at the site of cryotherapy; no volume reduction or shrinkage was reported (Figure 2D–F).

### 2.3. Keloid-Derived Fibroblasts Show Enhanced Proliferation and Migration Activity Following Treatment with −79 °C Spray-Type CryoPen™

**Figure 3:**
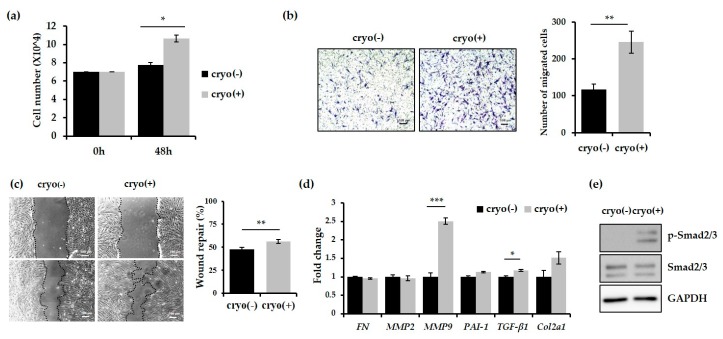
Cryotherapy increases the migration and proliferative activity of keloid dermal fibroblasts. (**a**) The comparison of the proliferative activity of the dermal fibroblasts from keloids before and after cryotherapy. Cell proliferation was promoted in the fibroblast treated with cryotherapy. The cell number was estimated at 48 h after cell seeding; (**b**,**c**) The migration activity of dermal fibroblasts was increased following cryotherapy. The migration of the cells was analysed by the transwell assay (**b**) and wound-healing assay (**c**); (**d**) qRT-PCR experiments were performed to evaluate the mRNA levels of fibronectin, MMP2 (Matrix metalloproteinase 2), MMP9, PAI-1 (Plasminogen activator inhibitor-1), TGF-β1, and Col2a1 (Collagen Type II Alpha 1) in fibroblasts derived from the dermis under the cryotherapy-treated (+) or untreated (−) keloids. MMP9 and TGF-β1 were significantly increased in the fibroblast treated with cryotherapy as compared with controls; (**e**) Immunoblot analysis of phosphorylated Smad2/3. Total Smad2/3 and GAPDH were used as the loading controls. Error bars represent the standard error from three repeated experiments. * *p* < 0.05, ** *p* < 0.01, and *** *p* < 0.005 (Student’s *t*-test).

We first compared the proliferation of dermal fibroblasts isolated from deep tissues of cryotherapy-treated or untreated keloids. As shown in Figure 3a, the number of dermal fibroblasts treated with cryotherapy was significantly higher than the untreated fibroblasts at 48 h after cell seeding, indicating that cryotherapy promotes proliferation of dermal fibroblasts. We compared the migration ability of dermal fibroblasts using the transwell assay. To exclude the effect of proliferation on the migration, the assay was performed with cells pretreated with mitomycin C, which causes cell cycle arrest. In comparison with the untreated fibroblasts, those treated with cryotherapy showed more than two-fold increase in their migration abilities (Figure 3b). Furthermore, fibroblasts subjected to cryotherapy showed significantly enhanced migration ability in the wound-healing assay (Figure 3c). Taken together, these findings indicate that the treatment of keloids with −79 °C spray-type CryoPen™ results in an increase in the proliferation and migration activity of fibroblasts.

### 2.4. Expression of MMP9 and TGF-β1 was Increased in Keloids after Treatment with −79 °C Spray-Type CryoPen™

The expression of MMP9, which promotes cell migration ability by converting various cytokines and chemokines into their more active forms, 12–14 was increased by more than two-fold following cryotherapy (Figure 3d). In addition, the expression of TGF-β1 and level of Smad2/3 phosphorylation were significantly increased by cryotherapy (Figure 3d,e).

### 2.5. Cryotherapy-Treated Keloids Showed More Fibrosis and Extensive Lymphoplasmacytic Infiltration

**Figure 4:**
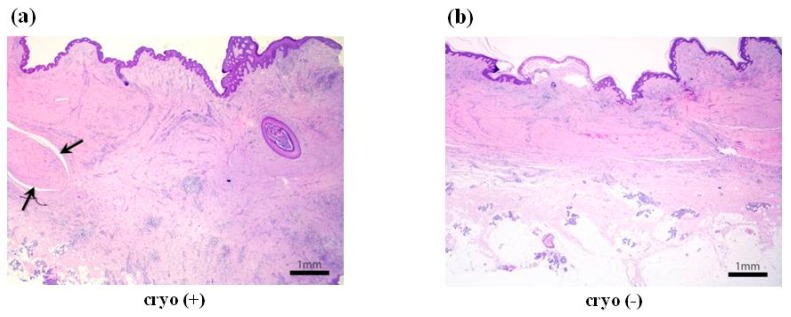
Histologic findings of keloids pretreated and untreated with cryotherapy. (**a**) Cryotherapy-treated keloid showed extensive lymphoplasmacytic infiltration characterised by thicker fibrosis and occasional “proliferating core collagen” (**black arrow**) in the deeper part (H&E stain, ×12.5); (**b**) The untreated keloid showed less inflammation and less surface-parallel fibrosis without core collagen (H&E stain, ×12.5).

The result of the molecular analysis was further confirmed by the pathologic examination. We found that keloids treated with cryotherapy showed extensive lymphoplasmacytic infiltration characterised with thicker fibrosis and occasional “proliferating core collagen” in the deeper part. On the other hand, untreated keloids showed relatively less lymphoplasmacytic infiltration and thinner, surface-parallel fibrosis without “proliferating core collagen” (Figure 4). Thus, the treatment of keloids with −79 °C spray-type CryoPen™ induces fibrotic incidence as compared with the untreated keloid tissues.

## 3. Discussion

Keloids are a result of an overgrowth of a dense fibrous tissue that develops after trauma of skin, and cryotherapy is known as an efficient and effective method for the treatment of keloids. However, our clinical experience of treating keloid patients with −79 °C spray-type cryotherapy protocol as a monotherapy has made us doubt the efficacy of this therapy in keloid treatment. 

More than 2 weeks after cryotherapy, the lesion reverted to its primary status and required additional cryotherapy (Figure 2C). In the severe case of keloids located at the upper arm, cryotherapy was ineffective and caused skin erosion at the site of cryotherapy; no volume reduction or shrinkage was observed (Figure 2D–F). These results imply that −79 °C spray-type cryotherapy may be effective only for the treatment of mild keloid and to destroy superficially located fibroblasts; the rapid recurrence of the symptom may be possibly due to the compensatory stimulation of the deep keloid tissues located under the cryotherapy-treated superficial keloid tissues.

We performed in vitro analysis of the cellular activity of the keloid-derived fibroblasts to understand the therapeutic effect of −79 °C spray-type cryotherapy. Dalkowski et al., used an experimental model for controlled cell freezing in vitro to simulate the effect of cryotherapy on keloid fibroblasts [17]. In our opinion, freezing cell may lead to mechanical destruction rather than actual physiologic effect in tissues. We also believed that superficial tissues, including epidermis and upper dermis of keloid, may be affected by the cryotherapy-mediated mechanical destruction. Hence, we harvested deep keloid tissues (at least 5 mm deep) from the epidermis for molecular analysis. Our results indicate that fibroblasts derived from the cryotherapy-treated keloids showed significantly enhanced proliferation and migration ability (Figure 3a–c). In addition, the mRNA expression of MMP9 and TGF-β1 was increased (Figure 3d) and the level of Smad2/3 phosphorylation was significantly up-regulated following cryotherapy (Figure 3d,e). Contrary to the general expectation that cryotherapy reduces the migration or proliferation ability of keloid fibroblasts through the inhibition of collagen synthesis [17], molecular analysis revealed that the proliferation and migration abilities of keloid fibroblasts from the deep tissue were significantly enhanced after cryotherapy, possibly due to the overexpression of MMP9 and activation of TGF-β signalling pathway.

Our current findings contradict the results demonstrating a significant reduction in TGF-β1 expression in the keloid tissue after cryotherapy by Awad et al., suggesting that a few sessions of cryotherapy normalised the abnormal collagen structure and reduced fibroblast proliferation by suppressing TGF-β1 expression [18]. The major discrepancy between these observations and those reported in our study arises from the quality of device used. While Awad et al., used the hand-held device (Brymill cryogenic system, CRY–AC, Ellington, CT, USA) with −196 °C liquid nitrogen, we performed our study using −79 °C liquid nitrogen. Furthermore, we used the simple spray-type cryotherapy as compared with the needle type cryotherapy used in their study.

However, Har-Shai et al. [19,20] reported successful outcome with intralesional cryosurgery treatment. Their technique resulted in major changes in collagen structure and organization including reduced the number of proliferating cells, of myofibroblasts and of mast cells. This means that intralesional cryosurgery effectively irradicates “proliferating core collagen” in keloids [21].

Taken together, we suggest that the monotherapy with −79 °C spray-type cryotherapy is ineffective for the treatment of thick keloids and should be used in the combination with intralesional injection of corticosteroids or botulinum toxin A to prevent any compensatory stimulation of deep keloid tissues. At present, we use this combination therapy as the first choice of treatment in keloid patients that are unwilling or unsuitable for any surgical treatment. However, further studies should be performed to optimise the cryotherapy protocol to provide patients and physicians with the best possible management of keloids.

## 4. Materials and Methods

### 4.1. Inclusion and Exclusion Criteria and Study Design

The current study was approved by the institutional review board of the CHA University (2017-03-051; 10 April 2017). Patients with keloids who presented to the outpatient clinic were included in the study based on the following criteria: (1) the scar was elevated and extended beyond the dimensions of the initial injury site or lesion; (2) patients were older than 18 years; (3) surgical excision was scheduled; (4) patients received no additional treatment or medication during the study and prior to surgical excision; and (5) patients signed up for the data use agreement as a basis to the clinical study. Patients were excluded from the study if they were unavailable for follow-up or wanted to stop cryotherapy treatment for any reason. Patients who had received any additional adjuvant therapy during the treatment were also excluded from the study. A total of ten keloids on six patients (all females) were included in this study and all keloids showed deep thickness. The detailed information of the cases is listed in Table 1.

### 4.2. Cryotherapy Protocol

All procedures were performed with −79 °C hand-held spray-type CryoPen™ (L&C BIO, Gyeonggi-do, Korea) without anaesthesia. Each keloid lesion was evenly divided into untreated area (C−) and treated area (C+). The C+ area was subjected to only two freeze-thaw cycles of spray-type cryotherapy. This treatment was repeated after an interval of 2 weeks. Eventually, the C+ lesion was treated with four sessions of cryotherapy over a period of 8 weeks prior to surgical excision. We then excised lesions completely, including a full layer of dermis until we noticed bleeding of the underneath subcutaneous tissue. The bleeding was controlled with bipolar electrocoagulator. The 3-mm thick and at least 5-mm-deep dermal keloid from the epidermis was used for primary cell culture, while the adjacent full layer from the epidermis to reticular dermis was used for pathologic examination (Figure 1). We closed wounds with an appropriate approximation using 5–0 nylon interrupted sutures. When primary closure was not possible, we covered the wound with full thickness skin graft from the inguinal area, fixed it with 5–0 black silk sutures, and a tie-over dressing was done.

### 4.3. Primary Cell Isolation, in Vitro Culture, and Cell Proliferation Assay

The fibroblasts were isolated from the dermis under the surgically excised and cryotherapy-treated or untreated keloids. The dermis was cut into approximately 5 mm^3^ pieces. The epidermis and lipid layer were removed with 2% dispase II (Sigma, St. Louis, MO, USA) and the connective tissue was digested in 0.5 mg/mL collagenase A (Sigma) at 37 °C for 3 h using a water bath. The digested solution was filtered through 70 µm strainer (BD biosciences, San Diego, CA, USA). The cell pellets were resuspended in, and washed with, 1× Dulbecco's phosphate-buffered saline (Gibco, Gaithersburg, MD, USA). The cells were cultured in Dulbecco’s modified Eagle’s medium (DMEM) medium (Gibco) supplemented with 10% fetal bovine serum (FBS, Gibco) and 1% penicillin/streptomycin (Gibco) at 37 °C and 5% CO_2_. The medium was replaced every 2–3 days and the cells were subcultured at 70–80% confluency. All experiments were performed with cells at passage 3. For proliferation assay, cells were cultured at a density of 7.0 × 10^4^ cells in a 60 mm^2^ dish. After 48 h, cells were detached with 0.05% trypsin/ethylenediaminetetraacetic acid (EDTA; Gibco) and the cell number was estimated with a haemocytometer.

### 4.4. Wound-Healing and Transwell Assay

We performed the wound-healing and transwell assay to compare the cell migration activity. For the wound-healing assay, 7.0 × 10^4^ cells were cultured in each well of 12-well plates. After 24 h, each well was treated with mitomycin C (1 mg/mL) for 2 h and scratched using yellow tips. The wounds were observed 24 h after scratching. For the transwell assay, 2.0 × 10^4^ cells were added to the upper well of a transwell plate (Corning Inc., Corning, NY, USA). The lower portion of the well contained DMEM supplemented with 10% FBS as a chemoattractant. After incubation for 24 h, the medium and cells present in the bottom chamber were removed; the cells that migrated to the bottom chamber were fixed in 4% paraformaldehyde (Santa Cruz Biotechnology Inc., Santa Cruz, CA, USA) and stained with crystal violet for 15 min. The migrated cells were counted in random fields using ImageJ (Bethesda, MD, USA: U. S. National Institutes of Health).

### 4.5. Quantitative Real-Time Polymerase Chain Reaction (qRT-PCR)

Total RNA was extracted using TRIzol (Invitrogen, Carlsbad, CA, USA) and 1 µg of complementary DNA (cDNA) was synthesised using the LeGene Express 1st Strand cDNA Synthesis System (LeGene Biosciences Inc., San Diego, CA, USA) according to manufacturer’s instructions. qRT-PCR analysis was performed using the synthetic cDNAs and TOPrealTM qPCR 2× PreMIX (Enzynomics, Daejeon, Korea). The expression of the target genes was normalised against that of glyceraldehydes 3-phosphate dehydrogenase (GAPDH). The PCR primers are listed in Table 2.

### 4.6. Western Blotting

Cells were harvested with phosphate-buffered saline (PBS) and lysed in the tissue lysis buffer (20 mM Tris-base [pH 7.4], 137 mM sodium chloride [NaCl], 2 mM EDTA, 1% Triton X-100, 25 mM β-glycerophosphate, 2 mM sodium pyrophosphate, 10% glycerol, 1 mM sodium orthovanadate, 1 mM benzamidine, and 1 mM phenylmethylsulfonyl fluoride). Total cell extracts were separated by sodium dodecyl sulfate polyacrylamide gel electrophoresis, transferred onto polyvinylidene fluoride membranes (BIORAD), and blotted with antibodies against Smad2/3 (Cell Signaling Technology, Beverly, MA, USA) and phospho-Smad2/3 (Cell Signaling Technology). Immunoreactivity was detected with enhanced chemiluminescence (Amersham).

### 4.7. Pathologic Study

Microscopic examination was performed for the cryotherapy-treated and untreated keloids using routine formalin-fixed, paraffin-embedded tissue process and hematoxylin-eosin staining. The excised keloids were fixed in 10% buffered formalin, and totally embedded in paraffin blocks after routine preparation process. Then, 7 µm-thin sections were subsequently stained by hematoxylin and eosin (H&E) for usual microscopic examination. The slides were assessed by pathologist (Yosep Chong).

## 5. Conclusions

Cryotherapy with the spray-type device is a relatively quick and minimally invasive technique, which may not necessitate local anaesthesia. However, we show that −79 °C hand-held spray-type cryotherapy is ineffective as a monotherapy and highlight the need for its use as a combination therapy with intralesional corticosteroids or botulinum toxin A to achieve favourable results for the treatment of keloids.

## Figures and Tables

**Table 1:** Baseline patient demographics in this study.

No. of Cases (*n*)	Age (Years)	Gender	Skin Type (Fitzpatrick)	Duration of Scar (Years)	Scar Location	Cause	Sessions (*n*)	Interval (Weeks)	No. of FTCs (Freeze-Thaw Cycle) (*n*)
1	21	F	F III-IV	7	Upper arm	spontaneous	4	2	2
2	21	F	F III-IV	7	Chest wall	spontaneous	4	2	2
3	21	F	F III-IV	7	pubis	spontaneous	4	2	2
4	21	F	F III-IV	7	Axilla	spontaneous	4	2	2
5	56	F	F I-II	30	chest wall	infection	4	2	2
6	56	F	F I-II	35	Shoulder	Vaccination	4	2	2
7	26	F	F I-II	0.5	Trochanter	Surgery	4	2	2
8	33	F	F I-II	2	Lobule	Piercing	4	2	2
9	21	F	F I-II	3	Helix	Piercing	4	2	2
10	17	F	F I-II	3	Helix	Piercing	4	2	2

**Table 2:** Quantitative real-time PCR primers.

No.	Gene	Direction	Sequences (5’ to 3’)
1	*GAPDH*	Forward	ACCACAGTCCATGCCATCAC
		Reverse	TCCACCACCCTGTTGCTGTA
2	*fibronectin*	Forward	CAGTGGGAGACCTCGAGAAG
		Reverse	TCCCTCGGAACATCAGAAAC
3	*TGFβ1*	Forward	GGACACCAACTATTGCTTCAG
		Reverse	TCCAGGCTCCAAATGTAGG
4	*MMP2*	Forward	ATGACAGCTGCACCACTGAG
		Reverse	ATTTGTTGCCCAGGAAAGTG
5	*MMP9*	Forward	ATTCAGGGAGACGCCCATTT
		Reverse	CTGCGT TTCCAAACCGAGTT
6	*col 2a1*	Forward	GGGAGTAATGCAAGGACCAA
		Reverse	ATCATCACCAGGCTTTCCAG

## Abbreviations

DMEM	Dulbecco’s modified Eagle’s medium
GAPDH	Glyceraldehyde 3-phosphate dehydrogenase
MMP9	Matrix metallopeptidase 9
PBS	Phosphate-buffered saline
qRT-PCR	Quantitative real-time polymerase chain reaction
TGF-β1	Transforming growth factor β1
